# A Multifunctional Capsule-like Puncture Biopsy Robot for the Gastrointestinal System

**DOI:** 10.3390/mi16050589

**Published:** 2025-05-18

**Authors:** Xinmiao Xu, Jinghan Gao, Dingwen Tong, Yiqun Zhao, Xinjian Fan, Wanning Ge

**Affiliations:** 1 School of Future Science and Engineering, Soochow University, Suzhou 215222, China; 2262402039@stu.suda.edu.cn (X.X.); 2362405061@stu.suda.edu.cn (J.G.); 2129401038@stu.suda.edu.cn (D.T.); 2129401067@stu.suda.edu.cn (Y.Z.); 2 School of Mechanical and Electrical Engineering, Soochow University, Suzhou 215131, China

**Keywords:** puncture biopsy robot, micro-electromagnetic actuator, thermal hemostasis, targeted drug delivery, submucosal tumors

## Abstract

Gastrointestinal submucosal tumors (SMTs) are difficult to diagnose accurately due to their deep location and the limitations of traditional biopsy tools. To address these issues, we propose a multifunctional capsule-shaped puncture biopsy robot (PBR) with capabilities for tissue sampling, thermal hemostasis, and multi-stage drug delivery. The PBR measures 27 mm in length and 13 mm in diameter, integrating a micro-scale electro-permanent magnetic system with a 60-turn dual-layer coil (wire diameter: 0.6 mm) to drive an 8 mm-depth puncture needle. A graphene–carbon nanotube composite heating film enables rapid and safe temperature elevation, achieving effective hemostasis and triggering sequential drug release using paraffin-based phase-change materials. Heating remains within the clinical safety range. Experiments demonstrated successful tissue penetration, precise magnetic control, and reliable staged pigment release simulating drug delivery. Tests on an ex vivo porcine stomach confirmed adaptability to irregular gastric surfaces. This compact PBR provides an integrated and minimally invasive approach to both the diagnosis and treatment of gastrointestinal lesions.

## 1. Introduction

Gastric cancer is one of the most common malignancies worldwide, with gastrointestinal cancers accounting for approximately one-quarter of global cancer incidence and one-third of cancer-related deaths [1,2]. Although both the incidence and mortality rates have declined in recent years [3], gastric cancer remains the fifth most commonly diagnosed cancer and the fifth leading cause of cancer-related death globally, according to GLOBOCAN 2022 [4]. In 2022, there were over 968,000 new cases and nearly 660,000 deaths related to gastric cancer [5]. The survival rate for stage I gastrointestinal cancers is significantly higher than for stage V, highlighting the critical importance of early diagnosis [6].

Currently, endoscopy remains the primary diagnostic approach for gastric cancer. Flexible endoscopes equipped with imaging systems allow for the real-time visualization of gastrointestinal structures and facilitate early tumor detection (T1; AJCC). However, this procedure is associated with discomfort and potential complications. To improve patient compliance, capsule robots (CRs) have emerged as a promising alternative due to their miniature size, which reduces both physical discomfort and psychological stress [7,8]. While CRs can detect tumors and are well tolerated by patients [9,10,11,12], they are unable to perform tissue biopsies, often necessitating a second invasive procedure. This prolongs diagnostic timelines and increases healthcare costs. Thus, the development of capsule-based biopsy technology capable of acquiring tissue in situ is of significant clinical value.

Tissue biopsy remains a cornerstone of modern diagnostics, especially in oncology, where the microscopic examination of tissue samples provides definitive information on cellular and molecular pathology [13,14,15]. Technological advances in biopsy procedures have improved diagnostic accuracy and therapeutic outcomes [16]. Conventionally, tissue acquisition relies on percutaneous needle insertion guided by radiologists [17,18,19], while robotic-assisted systems with high-stiffness arms have demonstrated enhanced stability and positioning precision [20,21,22]. In recent years, research has increasingly focused on in vivo gastrointestinal biopsy robots that can directly extract tissue from suspected lesions.

Several miniature biopsy robots have been proposed [23,24,25,26,27]. Song et al. developed a biopsy capsule robot (BCR) with a novel energy-storing spiral spring and gear-driven cutting blade for soft tissue laceration, minimizing tissue tearing [28]. Le et al. designed a magnetically actuated clamping module capable of precise targeting, though the unstable magnetic force could damage tissues during sampling [29]. Yim et al. proposed a magnetic BCR deploying u-shaped microgrippers that self-fold and retrieve tissue samples [30]. Chen et al. developed a probe-type device powered wirelessly and navigated via double-balloon-enteroscopy-style locomotion [31]. Simi et al. incorporated a magnetic torsion spring that enables blade deployment upon magnetic field removal [32]. Although these mechanisms can acquire samples, most operate only at the surface level and cannot access deeper tissues. Moreover, microgripper-based devices exhibit low sample yield (~3%) [33], and clinicians may prefer targeted biopsies over stochastic ones. A critical limitation shared by all prior designs is their inability to retrieve deep-layer samples, often failing to detect submucosal tumors (SMTs) [30,34,35,36], thereby reducing diagnostic accuracy.

Consequently, efforts have shifted toward needle-based miniature biopsy robots [37,38,39,40]. Lewen et al. proposed a soft robot with integrated tip steering and needle deployment for lung biopsies, actuated via radially expanding soft actuators [41]. Son et al. introduced a magnetically actuated capsule endoscope (B-MASCE) for fine-needle aspiration, guided by a Sarrus linkage and driven by an internal permanent magnet [42]. However, these systems often suffer from limited actuation stability and precision. Magnetic actuation has emerged as a promising strategy due to its wireless operation, biocompatibility, and high control resolution, offering multi-degree-of-freedom manipulation with minimal tissue interference. Ye et al. developed a magnetically actuated biopsy capsule robot (MABC) that navigates the gastrointestinal tract and performs tissue acquisition under a gradient magnetic field generated by an external electromagnetic actuation (EMA) system [43]. Hoang et al. designed a wireless capsule endoscope driven by an EMA-controlled screw mechanism to perform tissue sampling [24].

There are various actuation methods for micro-robotic end-effectors, including electric actuators [44], ultrasonic/piezoelectric actuators [45], pneumatic actuators [46], hydraulic actuators [47], and electromagnetic actuators [48], as well as Bowden cables [49] and chain drives [42] (reference numbers based on the original version). Among these, magnetic actuation is particularly advantageous due to its fast response, transparency to biological tissues, relative safety, good controllability, and strong penetration capability [50]. It serves as a powerful means for remote actuation and wireless control of magnetic devices, especially in the development of soft robotics and magnetic navigation technologies. Chen et al. reported a novel magnetically actuated hollow continuum microrobot using silicone as the primary structural material, which enables soft deformation and shows great potential in next-generation minimally invasive therapies [51]. Hoang et al. introduced a robotic dual-electromagnet actuation (DEMA) system capable of simultaneously actuating and localizing a magnetic capsule endoscope [52]. Lin et al. proposed a new magnetic continuum robot (MCR) with multi-mode control, which utilizes oppositely magnetized magnets and a hollow elastic tube, resulting in a larger reachable workspace and improved flexibility [53].

However, these systems rely heavily on high-powered external magnets for actuation, increasing control complexity and energy consumption, while limiting sampling depth and efficiency. To overcome these limitations, replacing external magnets with micro electromagnetic actuators offers a compact and efficient alternative, significantly reducing system footprint while improving spatial utilization, control accuracy, and clinical adaptability. Furthermore, current biopsy robots generally lack bleeding control mechanisms, despite the fact that fine-needle aspiration can lead to mucosal bleeding in approximately 8.2% of cases, especially for submucosal lesions in the gastrointestinal tract [54,55]. Integrating thermal hemostasis into the biopsy procedure can enable single-session tissue sampling with minimized bleeding risks.

Meanwhile, the precise locomotion of robots within the gastrointestinal (GI) tract enables targeted drug delivery to specific sites [56,57,58,59,60,61]. For instance, Tian et al. developed a microrobot system for delivering enzyme-responsive hydrogels to inhibit triple-negative breast cancer, achieving site-specific drug release with minimal off-target effects [62]. Such targeted delivery strategies significantly enhance drug utilization efficiency by concentrating therapeutic agents exclusively on intended tissues while minimizing side effects on surrounding areas [63].

However, current capsule robots still face several limitations in drug delivery applications. Mapara et al. noted that achieving the accurate localization and navigation of capsule robots within the gastrointestinal tract remains a challenge, particularly in the absence of active locomotion mechanisms [64]. Moreover, Wei et al. pointed out that the limited internal space of capsule robots restricts the drug payload capacity, making them unsuitable for therapies requiring large doses or continuous drug administration [65].

Therefore, the key scientific problem addressed in this study is how to realize a fully integrated, minimally invasive capsule system capable of precise tissue acquisition, effective in situ bleeding control, and multi-phase drug delivery in the confined, dynamic environment of the gastrointestinal tract.

**Figure 1:**
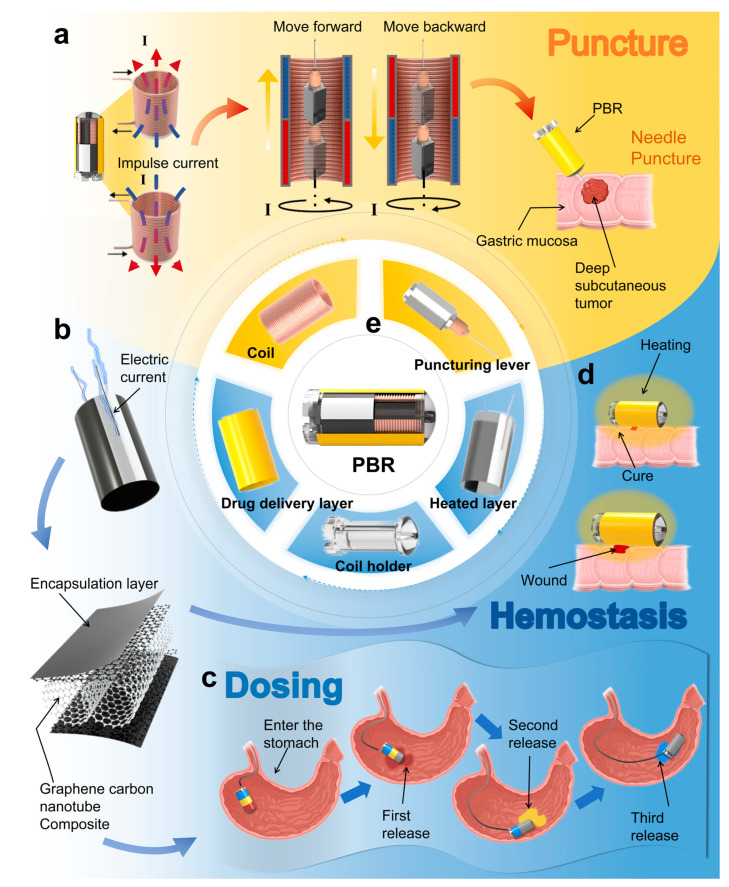
Structure, functional principles, and application scenarios of the proposed PBR system. (**a**) Puncture mechanism, actuation principle, and in situ tissue sampling demonstration. (**b**) Heating mechanism of the graphene–carbon nanotube composite thermal layer. (**c**) Three-stage segmented drug release process based on temperature-responsive phase-change materials. (**d**) Thermal coagulation hemostasis demonstration. (**e**) Structural composition of the puncture biopsy robot (PBR).

In this study, we propose a multifunctional capsule-shaped puncture biopsy robot (PBR) actuated by a distal electro-permanent magnetic system. As illustrated in Figure 1, the PBR is capable of executing puncture, targeted drug delivery, and thermal hemostasis for submucosal tumors, particularly in the stomach. Unlike previous designs dependent on external EMA platforms, our robot uses a miniature coil at the distal end to generate transient electromagnetic pulses, enabling needle deployment and retraction without external spatial constraints. A cuboid permanent magnet integrated with the needle allows for magnetic navigation and positioning. By applying sequential pulses, the robot performs stable needle advancement for tissue acquisition. For hemostasis, we incorporate a graphene–carbon nanotube composite heating film that provides precise thermal control to induce coagulative sealing of the puncture site. The same heating layer also enables multi-stage drug release by melting segmented paraffin-based phase-change materials for controlled therapeutic delivery. In this paper, we detail the design and fabrication of the PBR, characterize its puncture performance and heating safety, and demonstrate its functionality through tissue sampling experiments in ex vivo porcine stomachs.

## 2. Materials and Methods

### 2.1. Design of PBR

As shown in Figure 1e, the proposed puncture biopsy robot (PBR) primarily consists of the following four components: a puncture structure, a heating layer, a drug delivery layer, and a micro-electromagnetic actuation system. Under the precise control of the micro-electromagnetic system, the puncture structure enables accurate needle insertion and retraction. As illustrated in Figure 1a, to enhance the accuracy and controllability of the puncture process, we abandon traditional externally actuated magnetic mechanisms and instead utilize a transient current applied to a miniature coil to generate pulsed magnetic fields. These fields interact synergistically with an embedded permanent magnet, eliminating spatial constraints imposed by external control platforms. This design significantly improves operational flexibility and precision while maintaining compactness and high integration. Furthermore, under the guidance of an external gradient magnetic field, the permanent magnet allows the PBR to be accurately navigated to target locations, enhancing positioning accuracy and controllability. This coordinated design of the puncture mechanism and actuation system enables the robot to perform active targeting in complex and confined anatomical environments.

To minimize bleeding and alleviate pain during the puncture process, a mild thermal therapy strategy is employed, whereby heat ranging from 41 °C to 77 °C induces protein coagulation to seal the puncture site [66,67]. As shown in Figure 1b, a graphene–carbon nanotube (CNT) composite heating film is wrapped around the coil to form the heating layer [68,69]. Conductive silver paste is used to form electrodes on both sides, which are connected to power supply leads. To meet the low-voltage and high-output heating requirements, a dual-layer parallel heating film design is implemented. This configuration allows for rapid temperature rise and uniform heat distribution, enabling reliable thermal hemostasis. The temperature can be precisely controlled by adjusting the input voltage to maintain a stable therapeutic range.

Additionally, as depicted in Figure 1c, a segmented drug delivery module is integrated into the outer surface of the robot. Three types of paraffin-based phase-change materials (PCMs), each embedded with a different therapeutic agent, are longitudinally segmented and coated over the heating layer [70]. Upon reaching the target site, the heating layer triggers specific drug release by precisely reaching the melting thresholds of each PCM segment, thereby enabling multi-phase controlled drug release via a melt-diffusion mechanism. This design leverages the synergy between thermal actuation and phase transition materials, allowing the heating layer to serve the dual functions of achieving thermal hemostasis (Figure 1d) and initiating targeted drug delivery. Together, these functions form an integrated theranostic (therapy + diagnostic) control system within the PBR.

### 2.2. Preparation of PBR

The core components of the micro-electromagnetic actuation system include a miniature coil and a permanent magnet. To ensure sufficient puncture force, the number of coil turns was maximized. However, increasing the number of turns inevitably enlarges the coil’s cross-sectional area, which in turn increases the outer diameter of the robot, limiting its ability to pass through the esophagus and navigate within the gastrointestinal tract. Conversely, decreasing the cross-sectional area to increase the number of turns results in excessive heat generation, posing potential safety risks. Taking these factors into account, the coil was designed with an inner diameter of 5 mm, an outer diameter of 6.5 mm, and a total of 60 turns arranged in two layers of 30 turns each, using a single copper wire with a diameter of 0.6 mm.

For structural precision, a high-strength photosensitive resin (Tough 2000 Resin, Formlabs, Somerville, MA, USA) was used to 3D print the coil frame. Given the robot’s motion requirements in complex environments, the puncture channel was designed with a square cross-section (4 mm per side), and the front end of the frame was shaped in a capsule-like arc to facilitate smooth passage through the gastrointestinal tract. Four notches were designed in the gastric-facing region to house the coil, with two wires designated for powering the coil and two for the heating layer. To improve the robot’s flexibility in narrow anatomical spaces and overcome the rigidity of copper wires, the coil’s terminal connections were replaced with liquid metal (indium–tin alloy, melting point: 11 °C), which was encapsulated in a transparent soft silicone tube. This solution enhances flexibility and mobility while ensuring rapid heat dissipation and compatibility with high current levels.

The puncture mechanism employs a 23G biopsy needle (outer diameter 0.6 mm) commonly used in clinical applications, with an exposed length of 10 mm. The needle is fixed to the magnetic assembly via an adhesive-mounted needle seat. The magnetic assembly consists of two thin cylindrical NdFeB magnets and one cuboid NdFeB magnet. The cuboid magnet (5 mm × 5 mm × 8 mm, axially magnetized) couples with the transient magnetic field generated by the coil to actuate the needle forward and backward, while also enabling magnetic navigation and external actuation. The two cylindrical magnets (4 mm diameter, 1 mm thickness, radially magnetized) enable rolling motion under an external magnetic field, enhancing maneuverability and adaptability in complex anatomical environments.

Miniature robots intended for gastric applications must first enter the digestive tract through the oral cavity and ultimately reach the stomach. The average diameter of the adult esophagus is approximately 15–20 mm, which represents a primary constraint for the design and entry of such robots [71]. To ensure that the PBR can pass through the esophagus and enter the stomach without causing discomfort or damage to the digestive tract, the robot was designed with a final dimension of 27 mm in length and 13 mm in diameter. Its diameter is significantly smaller than the average esophageal diameter in adults, ensuring a safe and reliable operational range during locomotion.

For the heating and drug delivery modules, a composite structural strategy was implemented based on flexible electrothermal actuation and gradient phase-change-controlled drug release. The heating layer consists of a graphene–carbon nanotube composite film (flat dimensions: 52 mm × 22 mm), folded in parallel and wrapped into a semicylindrical shape (diameter: 14 mm) to conform to the robot’s body. Conductive silver paste (conductivity ≈ 66.7 S/m) is symmetrically coated along the axial direction to form electrode pathways, which are connected to polyimide-insulated copper wires (diameter: 0.4 mm). This configuration establishes a low-voltage (4.6–6.6 V) precision thermal control system with ±1.0 °C uniformity, outperforming conventional metallic heaters.

The drug delivery module comprises concentric annular layers of paraffin-based phase-change materials (PCMs) with gradient melting points (35–50 °C) that are axially segmented along the capsule surface. The melting points of the segments are 35 °C, 42 °C, and 50 °C from one end of the capsule to the other, with each segment’s thickness kept under 0.6 mm. By precisely controlling the heating temperature via the heating layer, the PCMs can be selectively activated to release the embedded drugs in a staged and programmable manner.

### 2.3. Drive and Motion Modes of PBR

Compared with electromagnets, permanent magnets offer stronger and more stable magnetic field outputs. Therefore, in this study, we selected a cubic permanent magnet with a side length of 25 mm as the external magnetic field source to achieve non-contact magnetic actuation of the robot. For the efficient estimation of the magnetic field vector at any spatial point, the permanent magnet was simplified as a magnetic dipole centered within its volume. The magnetic field $B\left(p\right)$ generated by the dipole at an arbitrary spatial point p can be described as follows:

(1) $$B\left(p\right)=\frac{\mu_{0}}{{4\pi \left\|r\right\|}^{3}}\left(\frac{3rr^{T}}{{\left\|r\right\|}^{2}}-I\right)M$$

where $\mu_{0}$ is the permeability of free space, r is the vector from the center of the magnet to the observation point, I is the identity matrix, and M is the magnetic moment of the dipole. Under this magnetic field, the force and torque exerted on the robot’s internal magnet can be described as follows:

(2) $$F=\left(m\cdot \;\nabla \right)B$$

(3) T=m×B

where B is the magnetic moment of the internal magnet and m is the local magnetic field strength. In the presence of a spatially varying external magnetic field (i.e., with a gradient), the robot is simultaneously subjected to magnetic force and torque, enabling combined translational and rotational control.

**Figure 2:**
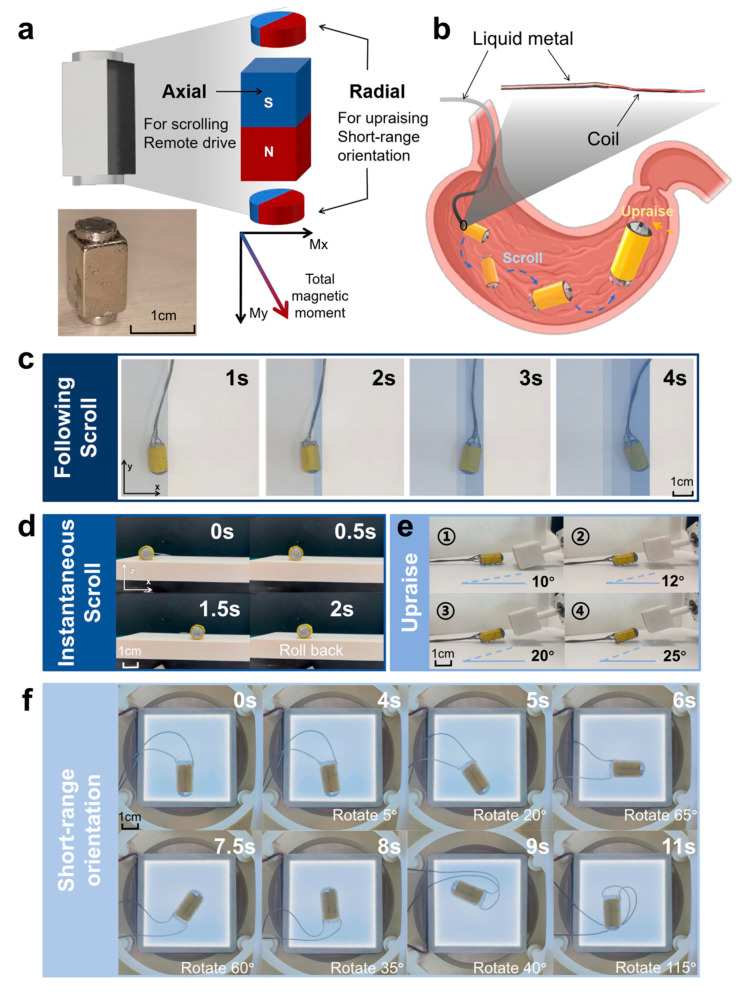
Actuation mechanism and locomotion modes of the PBR. (**a**) Photographs and schematic of the permanent magnet configuration and magnetization directions. (**b**) Locomotion strategy of the PBR inside the stomach and illustration of the tethering material. (**c**) Following scroll mode of the PBR. (**d**) Instantaneous scroll mode of the PBR. (**e**) Head pitch angle during puncture positioning. (**f**) Short-range directional rotation of the PBR in the planar magnetic field.

To enable effective locomotion and precise positioning within the gastric cavity, we designed a three-segment permanent magnet configuration, as shown in Figure 2a. The structure consists of two radially magnetized cylindrical magnets at both ends and one axially magnetized cuboid magnet in the center. This configuration ensures consistent and responsive magnetic behavior. As illustrated in Figure 2b, the PBR navigates by rolling along the mucosal surface toward the target site, after which the head can be elevated to adjust the puncture direction. To enhance maneuverability and flexibility, the tethering material was replaced with liquid metal, which provides both conductivity and bendability.

For planar motion, the PBR can perform two rolling modes: “following scroll” and “instantaneous scroll”. As shown in Figure 2c and Supplementary Video S1, the 25 mm cube magnet was positioned 20 cm below the robot and rotated at controlled angles and speeds via a stepper motor. At low rotational speeds, the robot followed a continuous and predictable trajectory. At higher speeds, the robot exhibited jumping or partial rollback behaviors due to incomplete flipping within each magnetic cycle (Figure 2d, Video S2). Nonetheless, effective displacement was still achieved, aided by mechanical inertia that allowed the robot to stabilize after transient disturbances.

During the puncture phase, as shown in Figure 2e and Video S3, the external magnet was positioned above the robot’s head, generating torque on the radially magnetized permanent sphere, enabling a pitch motion with a maximum elevation angle of 25°. Under a stable magnetic field, the head could remain suspended at a fixed angle, improving angular precision and adaptability within anatomically complex environments.

Finally, the PBR was placed within a three-axis Maxwell coil setup, where magnetic fields were applied in different directions to enable 360° planar rotation. As shown in Figure 2f and Video S4, the robot demonstrated stable and precise directional control, allowing for accurate orientation and puncture toward gastric tumors located in various internal positions.

## 3. Results

### 3.1. Puncture Distance Evaluation

The puncture motion of the PBR is based on a magnetic actuation principle. The robot body is constructed using a hollow cylindrical frame tightly wound with a copper micro-coil. This coil is wound in two layers with 30 turns per layer, connected via terminal leads at the distal end of the capsule, ensuring sufficient turns to enhance the generated magnetic field strength and stability. The micro-electromagnetic actuation system comprises a miniature coil and a cuboid-shaped NdFeB permanent magnet, which was designed to achieve efficient and precise remote actuation.

According to the magnetic effect of current, the application of a transient electric current through the coil rapidly generates an axial magnetic field. The intensity of the induced field varies with current magnitude, causing the embedded permanent magnet to move linearly along a predefined track inside the cylindrical frame. Due to the high coercivity and remanence of NdFeB, strong magnetic responses are achieved within a compact volume, enhancing the actuation force and control precision.

By applying continuous pulsed currents, the permanent magnet advances incrementally under magnetic force, resulting in a stable stepwise motion. Since the biopsy needle is rigidly attached to the front end of the magnet, this forward movement synchronously drives the needle into the target lesion. The puncture depth and speed can be adjusted by tuning the pulse frequency and amplitude, enabling precise penetration and efficient tissue acquisition. This non-contact electromagnetic mechanism eliminates the need for complex mechanical components, thereby enhancing the system’s safety, flexibility, and reliability while reducing spatial and procedural complexity.

Based on this puncture mechanism, we conducted experiments to evaluate puncture performance and determine optimal driving parameters. During testing, the magnet was positioned at the rear end of the robot, with the needle fully retracted into the capsule structure. The needle tip aligned with the outlet port was defined as the 0-position reference. Lightweight clay was used to simulate pathological tissue [43]. A single transient current was applied, and both the total and net advancement distances of the needle were recorded to assess the effectiveness of different actuation currents.

**Figure 3:**
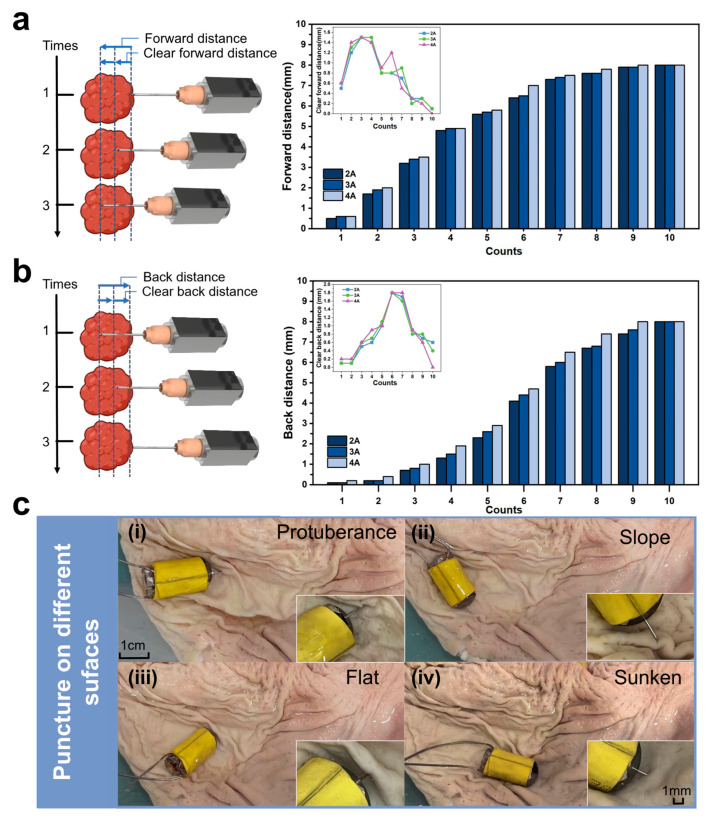
Evaluation of the puncture distance and surface adaptability of the PBR in ex vivo porcine stomach tissue. (**a**) Schematic of needle insertion: total insertion distance (SD: 2.608 mm (2 A), 2.587 mm (3 A), and 2.614 mm (4 A)) and net advancement (SD: 0.49 mm (2 A), 0.49 mm (3 A), and 0.52 mm (4 A)). (**b**) Schematic of needle retraction: total retraction distance (SD: 3.118 mm (2 A), 3.194 mm (3 A), and 3.273 mm (4 A)) and net withdrawal (SD: 0.57 mm (2 A), 0.53 mm (3 A), and 0.6 mm (4 A)). (**c**) Puncture demonstrations of the PBR on different tumor surface morphologies within a porcine stomach model: (**i**) protuberance; (**ii**) slope; (**iii**) flat; (**iv**) sunken.

As shown in Figure 3a, increasing the transient current from 2 A to 4 A resulted in greater magnetic excitation, leading to longer forward movement of the magnet along the track. However, due to structural limitations, the maximum exposed length of the needle was restricted to 8 mm, which became the limiting value for total puncture distance across all tested currents. Meanwhile, we calculated the standard deviation (SD) of the total puncture distance under three current levels, which were 2.608 mm (2 A), 2.587 mm (3 A), and 2.614 mm (4 A), respectively. The standard deviations (~2.6 mm) were highly consistent across all groups, indicating a similar degree of data dispersion under different current conditions. This uniformity reflects the stability of the experimental conditions and the repeatability of the results. According to prior studies [43], a minimum penetration depth of 5.1 mm is required for effective tissue acquisition, and our system successfully met this criterion. To analyze motion characteristics in more detail, the net puncture distance (i.e., the incremental advancement per pulse) was calculated. During the first three pulses, the net puncture distance exhibited an increasing trend. Although differences between current levels were minor, the curves converged toward a similar trend. Notably, the maximum net distance occurred at the third or fourth pulse. This can be attributed to the magnet reaching the coil center region when the needle is extended 3–5 mm. In this position, the coil’s induced magnetic field is concentrated axially, with minimal radial components, yielding optimal magnetic coupling and actuation.

To support safe retraction, the PBR is also capable of withdrawing the needle back into the capsule using the same actuation principle. Reversing the current polarity causes the magnet to retract, pulling the needle back and storing the biopsy sample internally for subsequent external analysis. As shown in Figure 3b, significant retraction is observed during the initial pulses, followed by a gradual reduction in retraction distance to approximately 0.3 mm per pulse. This trend mirrors the insertion phase and exhibits complementary behavior, validating the system’s bidirectional functionality. Interestingly, the maximum net retraction distance was observed during the sixth pulse, after which the displacement rapidly decreased.

A comparison of different current levels revealed that 2 A, 3 A, and 4 A currents followed similar retraction patterns. However, 2 A produced more variable results and showed insufficient force for effective retraction. While 4 A offered strong actuation, excessive heating due to liquid metal’s limited thermal dissipation posed risks of instability and potential tissue damage. In contrast, 3 A provided the most consistent and balanced performance, delivering sufficient force with acceptable thermal safety.

Taken together, the results suggest that a 3 A pulsed current yields optimal magnetic actuation performance in terms of efficiency, stability, and safety, and it is, therefore, selected as the recommended operating current for the PBR.

### 3.2. Demonstration of Puncture Scenes in Different Environments

In practical puncture procedures, the PBR must operate in diverse and complex anatomical environments. This is particularly true in the gastric cavity, where the mucosal surface is highly irregular, posing significant challenges for stable and effective biopsy. To ensure successful tissue acquisition under such conditions, the robot must exhibit strong adaptability and flexibility, enabling it to perform precise puncture operations on various lesion surface geometries and extract sufficient pathological samples.

To evaluate the PBR’s puncture performance across different environments, we simulated the following four representative mucosal surface morphologies commonly encountered during locomotion in the stomach: protuberance, slope, flat, and sunken surfaces. As shown in Figure 3c and Video S5, the robot was placed in an ex vivo porcine stomach under each of these surface conditions. Electric current was applied to activate the puncture mechanism, and the system’s ability to perform effective puncture across these geometries was tested.

As illustrated in Figure 3c(i–iv), the biopsy needle successfully extended and penetrated the mucosal surface under all four conditions, indicating that the PBR demonstrates excellent environmental adaptability. These results confirm the robot’s capability to perform reliable puncture operations in the anatomically complex and variable environment of the stomach.

### 3.3. Coil Temperature Evaluation

During the puncture operation, pulsed direct current is applied to the miniature coil to generate a transient magnetic field, which actuates the permanent magnet and thereby drives the biopsy needle attached to its front end to complete the tissue extraction. Prior to puncture, when the robot is navigated to the target site, a continuous direct current is supplied to the coil to maintain the needle in a retracted position within the capsule body, preventing unintended tissue injury. Although the applied DC current and voltage levels are relatively low and do not pose a direct safety risk in vivo, the thermal effect generated by the energized coil must not be overlooked. Specifically, miniature coils can produce considerable heat in short durations, especially when the wire diameter is small or the current is high. Similarly, liquid metal used as the flexible end-connection material to replace copper wires also contributes to heat generation during operation.

**Figure 4:**
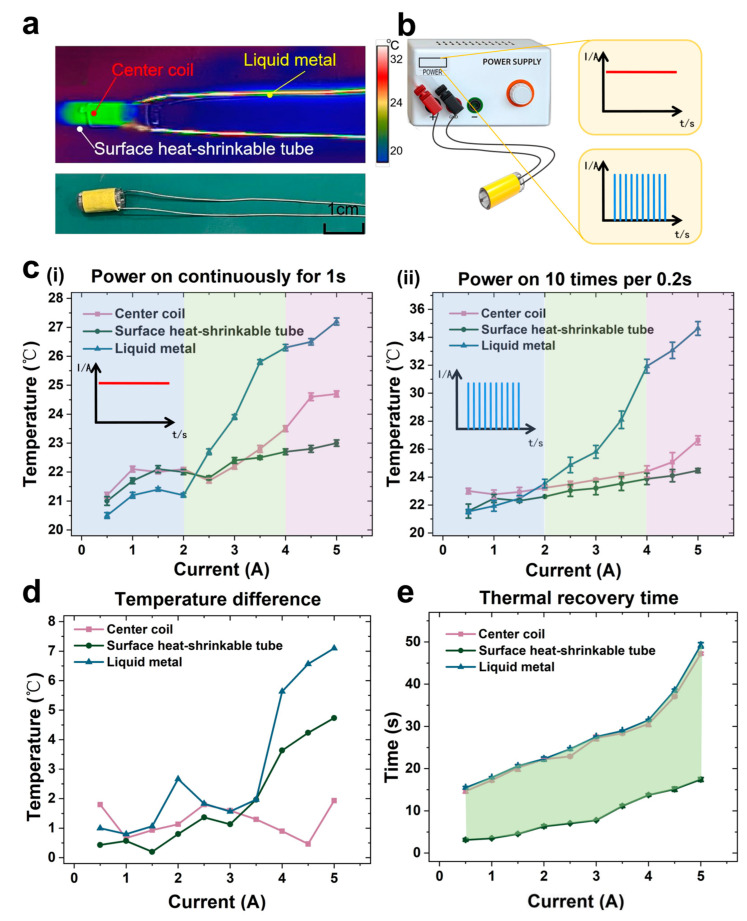
Thermal safety assessment of the micro-coil heating effect. (**a**) Photographs and infrared thermal images captured during safety evaluation using a thermal imaging camera. (**b**) Temperature comparison under two current input types: continuous current (1 s) vs. 10 pulsed currents (0.2 s interval). (**c**) Temperature profiles of the coil center, surface heat-shrink tubing, and liquid metal under different current conditions: (**i**) after 1 s of continuous current; (**ii**) after 10 pulsed currents with 0.2 s intervals. (**d**) Temperature increase difference between the two current input types. (**e**) Cooling time required after pulsed current input to return to the temperature observed under 1 s continuous current.

To assess the thermal safety of the PBR during actuation, we recorded temperature changes at different parts of the robot using a thermal imaging camera (HM-TPK20-3AQF/W, HIKMICRO, Hangzhou, China), as shown in Figure 4a. The heat generation and transfer characteristics of both the micro-coil and the liquid metal were evaluated under various DC current levels.

When electric current passes through a conductor such as a metallic coil, the internal resistance impedes electron flow, causing collisions between free electrons and metal ions. These interactions convert part of the electrical energy into thermal energy, increasing the temperature of the conductor. This process is governed by Joule’s law, shown as follows:

(4) $$Q=I^{2}Rt$$

where Q is the generated heat, I is the current, R is the resistance, and t is the conduction time. Heat generated by the coil is transferred radially through the surrounding silicone tube. This transfer can be described using Fourier’s law in cylindrical coordinates under steady-state conditions, shown as follows:

(5) $$T(r)=T_{s}+\frac{q}{4k}(R^{2}-r^{2})$$

where $T_{\left(r\right)}$ is the temperature at the radial position r, $T_{s}$ is the outer surface temperature, q is the internal heat generation rate, R and r are the outer and inner radius of the silicone tube, and k is its thermal conductivity. This model provides insight into the radial temperature distribution across the insulation layer.

Considering that the puncture process involves multiple, closely spaced activations, we first measured the thermal response after 1 s of continuous current application. Since the outermost heat-shrink tubing is in direct contact with human tissue during operation, we monitored the coil’s central temperature and the outer surface temperature of the tubing. The central temperature of the liquid metal component was also recorded.

At ambient temperature (19.5 °C), with a wire diameter of 0.8 mm and a dual-layer 30-turn winding configuration, the results of the 1 s continuous power tests are shown in Figure 4c(i). When the applied current was less than 4 A, both the coil center and the heat-shrink tubing exhibited similar, modest temperature rises (~4 °C). However, once the current exceeded 4 A, a steeper temperature increase (1.5 °C) was observed at the coil center, while the surface temperature of the tubing remained relatively stable. These results indicate safe thermal behavior at the tissue-contacting surface. Nevertheless, the liquid metal exhibited a much faster heating rate. Although its temperature remained below that of the coil and surface at low current levels, at 5 A, it showed a temperature rise of 8 °C within just 1 s. This suggests the need to avoid the prolonged application of high current during operation.

To evaluate thermal behavior under repetitive actuation conditions, which better simulate realistic puncture scenarios, we applied 10 pulsed current inputs with 0.2 s intervals and recorded the thermal response, as shown in Figure 4c(ii). Compared to the single 1 s pulse, all components exhibited greater temperature increases. At 5 A, the coil center and surface temperatures rose by 6.5 °C, while the liquid metal reached 35 °C, with a 15.5 °C rise. These elevated values resulted from limited dissipation time between pulses. Figure 4d shows the difference in temperature increase between the single-pulse and repeated-pulse conditions. At low current levels, the difference was minimal, while, at higher currents, the temperature rise became significant. Nevertheless, all observed temperatures remained within safe physiological limits, and the brief duration of exposure made thermal damage to tissue negligible.

However, to evaluate the potential long-term tissue damage caused by repeated thermal activation, we referred to current studies on cumulative thermal dose. Previous research has shown that the threshold for tissue damage depends not only on temperature but also closely on exposure duration, with a significantly increased risk of damage observed when the temperature exceeds 43 °C [72]. In our system, the heating duration for each activation is limited to less than 30 s to minimize the risk of thermal injury.

Finally, we assessed the cooling performance of each component after the repeated pulsed current tests. The time required for the temperature to return to levels observed after a single 1 s pulse was used as a benchmark, as shown in Figure 4e. The liquid metal and coil center shared similar slow cooling trends, while the surface heat-shrink tubing exhibited rapid cooling across all current conditions. At 5 A, the tubing returned to baseline temperature within 15 s, whereas the coil center and liquid metal required up to 50 s. The fast dissipation rate of the outer layer further ensures the operational safety of the robot within the human body.

### 3.4. Heat Hemostasis and Drug Release Temperature Evaluation

The structural design of the thermal hemostasis function module uses an innovative flexible electrothermal drive scheme. The core heating element consists of a carbon nanotube–graphene composite membrane. The carbon nanotube–graphene composite membrane thermal hemostasis module demonstrates multiple advantages through material synergy. Its unique three-dimensional thermal conductivity network gives very high heat transfer efficiency, combined with low voltage drive characteristics for fast thermal response. The flexible design supports large strain deformation and maintains the stability of the conductive network during dynamic operation; the precise temperature control technology realizes uniform heat generation and avoids the risk of local overheating. Superhydrophobic surfaces and biocompatibility certifications ensure safety in clinical applications [69,70].

**Figure 5:**
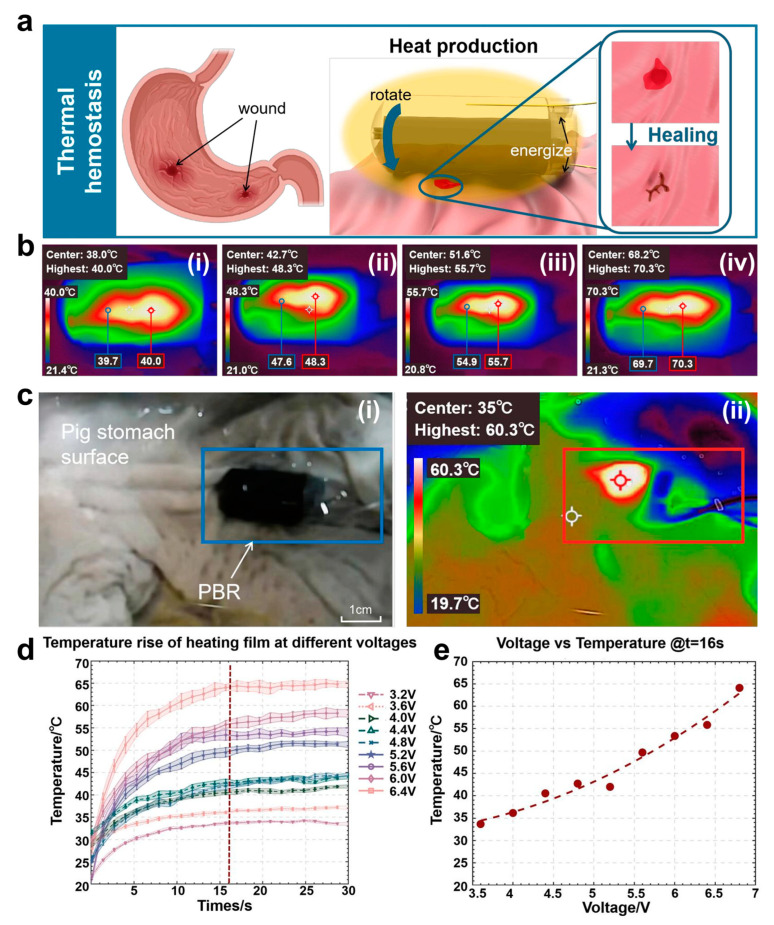
Explanation and evaluation experiment of the principle of heating hemostasis: (**a**) schematic diagram of the application of the thermal hemostasis function module. (**b**) from (**i**–**iv**), the constant pressure sources are respectively 3.6 V, 4.6 V, 5.6 V, and 6.6 V at the stable temperatures of the thermal hemostasis module. (**c**) actual heating process diagram: (**i**) original image; (ii) infrared thermal image. (**d**) heating and temperature rise conditions under different voltages. (**e**) relationship between steady-state heating temperature and voltage.

After the robot completes the puncture biopsy function, constant voltage electricity is applied to the electrothermal circuit. Thermal composite electric heating film output is hot enough to rapidly warm up to the temperature required for thermal hemostasis, so that some of the proteins in the wound after puncture are thermally denatured and coagulated, realizing the function of thermal hemostasis, as shown in Figure 5a,c, Video S6. In Figure 5c(ii), the red box indicates the actual position of PBR.

According to the experimental results of thermal hemostasis temperature evaluation shown in Figure 5d,e, the module demonstrated excellent temperature response characteristics and uniformity under constant voltage drive. As shown in the temperature–time curve of Figure 5d, when different voltages were applied, the temperature measurement point was rapidly warmed up to the target temperature from the room temperature (21.0 ± 0.2 °C) within 13 s, and the steady state temperature showed linear growth characteristics (33.4 °C→65.1 °C) as the voltage was stepped up from 3.2 V to 6.4 V (step 0.4 V). The steady-state temperature showed a linear growth characteristic (33.4 °C→65.1 °C), its high temperature responsiveness ensured timely thermal hemostasis after puncture, and the stability of the highest temperature also ensured the biosafety performance. As further verified by the thermal imaging data in Figure 5e, the temperature difference between the highest temperature point and the edge point of the heated area was maintained within ±1 °C (70.3 °C vs. 69.7 °C), which proved that the temperature uniformity of this composite film under the working voltage (temperature difference between the highest point and the edge of the temperature point was ≤2.0 °C) was significantly better than that of the traditional metal heaters (temperature difference of up to 8.5 °C), and the risk of tissue damage due to localized overheating was effectively circumvented The temperature difference was significantly better than that of traditional metal heaters (temperature difference of 8.5 °C), effectively avoiding the risk of tissue damage caused by local overheating. Under the critical voltage of 6.6 V, the temperature of the wound contact area was precisely stabilized at 70.0 ± 0.5 °C (as shown by the red markers in Figure 5b(iv)), which fully met the threshold requirement of protein thermal denaturation and coagulation.

Next, the heat release mechanism of the robot’s heating layer was utilized to further expand the functional outreach of the thermal hemostasis module, and a gradient multistage drug-controlled release system based on the phase-change material paraffin was innovatively constructed on the robot’s surface. The heating is carried out through the change of voltage, which triggers the slow release of the other three types of drugs sequentially.

**Figure 6:**
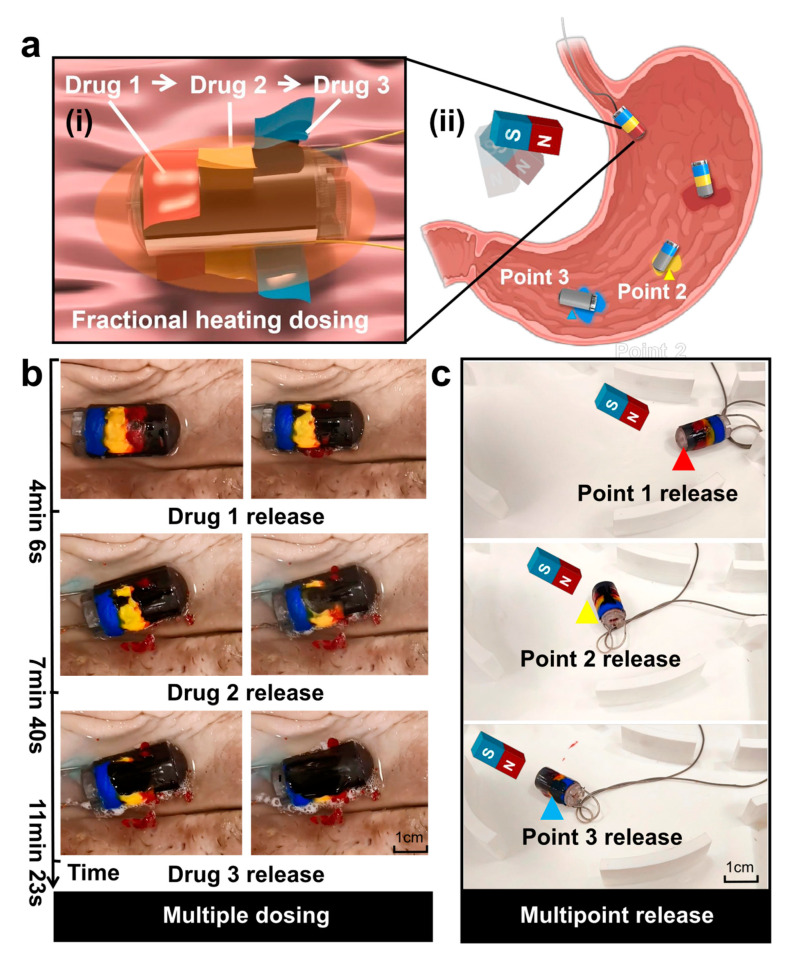
Design and experiment of PBR’s drug delivery function. (**a**) Schematic diagram of the robot’s drug delivery module: (**i**) heated segmental release; (**ii**) multipoint drug delivery. (**b**) Segmental drug delivery in the porcine stomach. (**c**) Fixed-point drug delivery in the stomach model.

As schematically illustrated in the drug delivery module of Figure 6a, three phase-change paraffin coatings (melting points of 35 °C, 42 °C, and 50 °C, respectively) are integrated on the robot surface, each loaded with a different functional pigment to simulate the drug release process. The temperature setting for the drug complies with clinical safety standards and the requirements for drug stability [73,74]. When the thermal hemostasis module is driven by a stepped voltage (4.6–6.6 V), the uniform temperature field (±1.0 °C) on its surface precisely triggers the phase transition of the target paraffin layer. As obtained from the experimental data in Figure 6b, the low-melting-point layer (35 °C) melts and releases the red pigment at a voltage of 40.0 °C at 3.6 V, the mid-layer (42 °C) triggers the release of the yellow pigment at 48.3 °C at 4.6 V, and the high-melting-point layer releases the blue pigment at 55.7 °C at 5.6 V. The high melting-point layer releases the blue pigment at 50 °C at 5.6 V. The melting point layer releases blue pigment, and the experiment shows that the layered isolation of the phase-change material is effective. Meanwhile, as shown in Figure 6c, the design drives the magnetic capsule robot to a specified position with a rotating magnetic field through the temperature-space triple control, which transforms the precise temperature control advantage of the thermal hemostasis module into a physical trigger for drug release, and realizes the function of drug administration in multiple times at different positions, as shown in Video S7.

To further validate the controllability of the multistage drug release system, quantitative experiments were conducted to measure the release efficiency and rate of each paraffin-encapsulated drug model. Each layer was embedded with 10 mg of functional pigment, and after thermal activation under specific voltage conditions, the amount of pigment released into the surrounding solution was determined using UV–Vis spectrophotometry at corresponding wavelengths (520 nm for red, 450 nm for yellow, and 600 nm for blue). The experimental results showed that the red, yellow, and blue pigments were released with efficiencies of 83.2%, 79.0%, and 81.0%, respectively, with corresponding release rates of 0.098 mg/s, 0.086 mg/s, and 0.091 mg/s, within time frames of approximately 85–92 s. These findings confirm that the thermal hemostasis module enables controlled and efficient phase-change-triggered drug release. The layered paraffin coatings not only provided thermal insulation between layers but also ensured temperature-specific release without premature activation or cross-layer interference, thereby demonstrating the feasibility of precise, staged drug delivery in both spatial and temporal dimensions.

## 4. Conclusions

In this study, we designed and fabricated a novel multifunctional capsule-shaped biopsy robot (PBR), which integrates a micro-electromagnetic-driven puncturing mechanism with a graphene-based composite heating membrane for hemostasis and controlled drug release. The system is capable of active in vivo puncturing, thermal hemostasis, and targeted drug delivery. The micro-electromagnetic-driven structure effectively overcomes the spatial limitations posed by traditional external magnetic control platforms, significantly enhancing the flexibility and precision of in vivo puncturing operations. The PBR can move to a designated area of the stomach by rolling under the guidance of an external strong magnetic field, and then perform fine-angle rotation to target suspected lesions for puncture. Furthermore, through thermal safety assessments and multi-scenario porcine stomach puncture experiments, the reliability and practicality of the system in terms of thermal control safety, environmental adaptability, and sample acquisition efficiency were verified. Finally, we measured the temperature increase effect of the external heating layer and demonstrated the three-stage multi-point drug delivery function achieved through multi-point driving. This study provides a novel minimally invasive solution for the early diagnosis and precise treatment of gastrointestinal tumors, with promising clinical application prospects.

In future work, we will conduct more rigorous thermal effect evaluation experiments within the stomach to enhance system safety and ensure that heat accumulation caused by repeated coil activation and sustained heating of the thermal layer does not lead to long-term tissue damage. We plan to focus on integrating clinically used chemotherapeutic agents into the PBR system. Meanwhile, we will further enhance the puncturing capability of the PBR and conduct experiments on real tumors or ex vivo gastric tissue.

## Data Availability

All data needed to evaluate the conclusions in this paper are present in this paper and the Supplementary Materials.

## Supplementary Materials

The following supporting information can be downloaded at: https://www.mdpi.com/article/10.3390/mi16050589/s1, Video S1: Following scroll; Video S2: PBR’s upraise; Video S3: Lnstantaneous scroll; Video S4: Short-range directional rotation; Video S5: Surface puncture of tumors of different shapes in the stomach; Video S6: Evaluation of thermal hemostasis temperature; Video S7: Administer the drug in segments at three points.
